# What Footwear Do People with Diabetes Mellitus Use? A Narrative Review

**DOI:** 10.3390/jcm14238529

**Published:** 2025-12-01

**Authors:** Raúl Carral-Sota, María Reina-Bueno, María del Carmen Vázquez-Bautista, Salomón Benhamú-Benhamú, Inmaculada Concepción Palomo-Toucedo

**Affiliations:** Department of Podiatry, Faculty of Nursing, Physiotherapy and Podiatry, Universidad de Sevilla, 41009 Seville, Spain; rcarral@us.es (R.C.-S.); mreina1@us.es (M.R.-B.); carmenvaz@us.es (M.d.C.V.-B.); benhamu@us.es (S.B.-B.)

**Keywords:** Diabetes Mellitus, ulcer, footwear, shoe, trauma, prevention, shoe adjustment, shoe fit

## Abstract

**Background/Objectives**: The prevention of foot ulcers is a priority for the preservation of the integrity of limbs in subjects with Diabetes Mellitus. Footwear is one of the main causes of ulceration regarding this chronic disease. An in-depth study of the influence of footwear is necessary for the establishment of a prevention strategy for foot injuries. This paper aims to identify the type of footwear used by patients with Diabetes Mellitus and to analyse its characteristics and fit to the foot. **Methods**: The scientific literature was retrieved from the PubMed, Web of Science, and Dialnet databases that covered publications from 2016 to 2025. The inclusion criteria accepted articles that focused on footwear fit and its relationship with ulceration in diabetic patients. **Results**: A total of 27 articles were selected for study. These articles describe the fitting and features of footwear for a correct adjustment for people with Diabetes. **Conclusions**: Most of the population with Diabetes Mellitus use poorly adjusted footwear. Incorrect length and width, as well as unsuitable features of the sole and upper, cause patients to become susceptible to the creation of ulcers.

## 1. Introduction

Diabetes Mellitus (DM) is a chronic, serious metabolic disorder characterised by persistent hyperglycaemia resulting from impaired insulin secretion, insulin action, or both. This dysfunction hinders the cellular uptake of glucose, thereby disrupting normal energy metabolism and storage. Current estimates indicate a global prevalence of DM in approximately 589 million adults aged 20 to 79 years, which represents 11.1% of the worldwide population [1].

Diabetes Mellitus entails a range of health complications, and particularly affects the lower limbs, including Peripheral Neuropathy (PN) and Peripheral Arterial Disease (PAD) [2]. Recently, the International Working Group on the Diabetic Foot (IWGDF) introduced the term Diabetes-Related Foot Disease, which encompasses all diabetes-related complications that affect the foot [3]. Due to sensory, motor, and autonomic neuropathies, patients experience alterations in protective sensation, deformities, and biomechanical abnormalities, as well as viscoelastic disturbances in the skin of the feet [4]. When these changes are compounded by trauma and varying degrees of peripheral vascular disease, they can lead to foot ulceration, with an increased susceptibility to infection, and result in a clinical condition known as Diabetic Foot [4]. It is estimated that up to 34% of diabetic patients develop a foot ulcer at some point in their lifetime [3].

According to the IWGDF, the use of inappropriate footwear (and walking barefoot) is one of the primary causes of trauma, which, in neuropathic feet under the aforementioned conditions and multiple risk factors, significantly increases the predisposition to the development of ulcers [5].

It is estimated that between 33% and 82% of individuals with DM wear improperly fitting footwear [6], which causes microtraumas that lead to excessive mechanical pressure and friction on the foot, potentially triggering stress ulcers of the skin and underlying tissues [7]. If the existing injury is compounded by the patient’s lack of awareness due to peripheral neuropathy and continued use of the same footwear, then the shoe becomes a perpetuating and aggravating factor of the ulcer [5], which carries a potential risk of infection and progression to amputation [7].

The literature provides a vast store of evidence regarding the importance of offloading in the management and healing of diabetic foot ulcers with a neuropathic component, and of the use of therapeutic footwear in the prevention of ulcers in these patients. However, adherence to these methods remains low among patients [8,9].

Prevention of ulceration is essential to reduce patient comorbidities and healthcare costs. It is therefore crucial to ensure proper footwear fit, together with the application of the appropriate materials and design adapted to the morpho-functional conditions of diabetic patients [7].

In this article, a narrative review of studies is conducted that focuses on the footwear fit and features that produce poor foot adjustments in people with Diabetes Mellitus.

## 2. Materials and Methods

### 2.1. Search Strategy

The study was initially designed to conduct a systematic review registered in Prospero CRD42024604216, to answer the question: “Is the footwear worn by people with Diabetes appropriate? Due to the lack of literature that met the inclusion criteria, the authors have decided to carry out a narrative review.

An extensive search was carried out between February and June 2025 in several databases, including PubMed, Web of Science, Dialnet, and the FAMA catalogue of the University of Seville. The Scopus database was not reflected since the PubMed database already encompassed the articles indexed in Scopus. Subsequently, in an additional search of the Web of Science database, two new studies were found that met the inclusion criteria.

To optimise the search, health-related descriptors relevant to the subject were used, along with free terms combined with Boolean operators, and in some cases, MeSH terms: “Diabetes Mellitus”, “Shoes”, “Ulcers”, “Diabetic foot” and “Prevention”. An advanced search tool was employed in all cases to design the search strategies. A specific strategy was developed for each database, which is given in detail in Table 1.

The aim of this review was to assess the existing evidence on the outcomes of fitting and footwear features that produce a poor adjustment in people with DM.

### 2.2. Selection Criteria and Study Selection

The selection was not limited to any specific article design, due to the insufficient number of publications for a systematic review to be conducted on the topic, and valuable information could have been lost if a limit had been imposed. This led us to carry out a narrative review, including articles with different levels of scientific evidence, published in English or Spanish, with a filter for the past 10 years.

The inclusion criteria for this review, therefore, encapsulated the following: articles addressing footwear fit and its correlation with the development of ulcers in patients with diabetes.

The exclusion criteria included: documents published before 2016, those written in languages other than English or Spanish, those that do not refer to footwear in adults and/or individuals with Diabetes Mellitus, and those related to therapeutic footwear prescribed for a specific pathological condition.

For the study selection process, two independent reviewers, R.C.-S. and S.B.-B., were appointed. Each reviewer conducted the selection process independently. In the initial stage, they screened the titles and abstracts of the shortlisted articles. Both reviewers then individually assessed whether the studies met the inclusion and exclusion criteria. Moreover, they separately evaluated the key characteristics of the studies to ensure strict adherence to the defined eligibility criteria.

In instances where disagreements arose regarding the inclusion of specific articles, a third reviewer (M.R.-B.) was consulted to carry out the final determination on the suitability of the article in question.

### 2.3. Data Extraction

In order to facilitate data extraction from the articles included in this review, Zotero 7.0.27 for Windows (Roy Rosenzweig Center for History and New Media, George Mason University, Fairfax, VA, USA) was employed for reference management, identification of duplicate records, and organisation of the bibliographic database. An Excel spreadsheet of Microsoft Windows 365 (Microsoft Corporation, Redmond, WA, USA) was created to systematically extract, categorise, and synthesise the information from each study. The inclusion or exclusion of each study was documented. Each reviewer maintained their own worksheet. Following the final assessment, the results were consolidated, and any discrepancies regarding article inclusion or exclusion were discussed. Initially, these discussions took place between the two primary reviewers, although in certain cases, the involvement of a third reviewer was required to reach a final decision.

Ultimately, two reviewers (R.C.-S. and I.C.P-T.) performed the complete data extraction for the articles selected subsequent to the second assessment. For this task, a Word document was utilised to record the identification and key characteristics of the studies (study type, number of participants, mean age, intervention, outcomes, etc.), thereby ensuring strict adherence to the eligibility criteria established for the review.

## 3. Results

### 3.1. Study Selection

**Figure 1:**
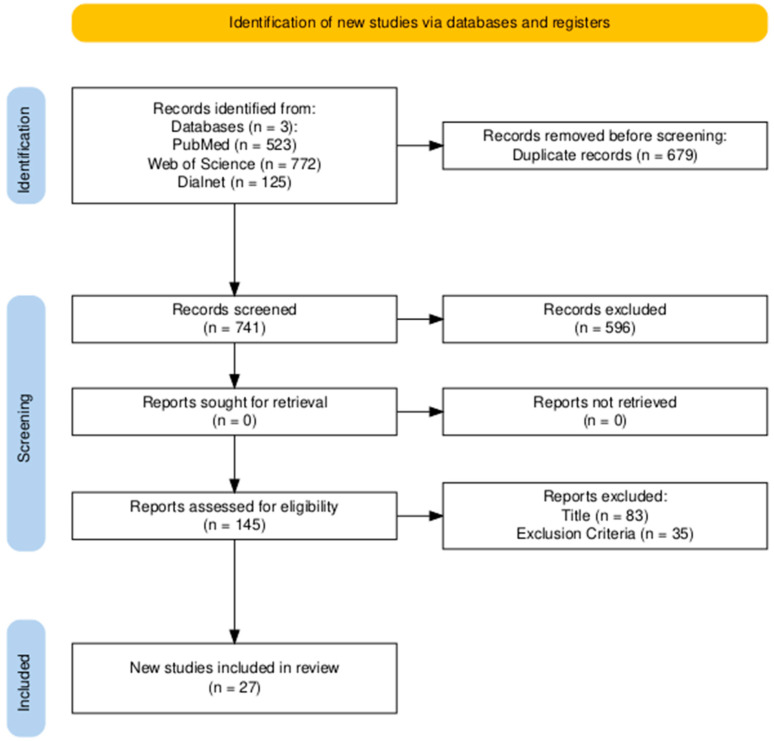
PRISMA flow diagram adopted for this review.

A total of 1420 articles were identified through the search. After having screened the titles and abstracts, a further 1275 articles were excluded. Full-text screening of the remaining 145 studies was then conducted, resulting in the 27 final inclusion of relevant articles. In cases where discrepancies arose between the two researchers, they discussed the articles to reach a consensus. Following the full-text review, 2 studies were rejected since they contained irrelevant information. Table 1 shows the search strategies, and Figure 1, the selection process of the studies included in this review.

### 3.2. Study Characteristics

Among the 27 studies included in the review, 3 were Systematic Reviews and Meta-analyses [10,11,12], 4 were Systematic Reviews [4,13,14,15], 1 was a Randomised Clinical Trial [16], 1 was a Case–Control Study [17], 8 were Observational Studies [7,18,19,20,21,22,23,24], 1 was a Qualitative Study [25], and 9 were Narrative Reviews [5,6,26,27,28,29,30,31,32]. Due to the different types of design, the levels of evidence vary widely. The main outcomes of each article are summarised in Table 2.

The scientific literature on footwear for patients with Diabetes Mellitus reveals a progressive consensus regarding specific design features that contribute to the prevention and reduction in plantar ulcers. First, rigid soles and rocker sole designs constitute the most frequent recommendation, appearing in approximately 43% of the reviewed studies. Bus et al. [13,29] and Premkumar et al. [17] highlight their capacity to decrease plantar pressure and prevent forefoot ulceration. López-Moral et al. [16,20] demonstrate that rigid soles reduce ulcer recurrence by 64% compared to semi-rigid soles. Collings et al. [10] and Ahmed et al. [14] provide systematic evidence that these soles reduce metatarsal head pressure by 30–50%. Lastly, Van Netten et al. [12] confirm that footwear with rigid soles decreases both the incidence of initial ulcers and their recurrence.

Secondly, toe-box spacing and internal shoe length are identified as critical factors in approximately 29% of the studies. Mohamed et al. [18] recommend an excess length of 1–2 cm beyond the foot, while Bus et al. [29] and Jones et al. [30] suggest an optimal range of 1–1.5 cm, accompanied by a sufficiently high toe box. Chicharro-Luna et al. [7] and Zhang et al. [23] add that the toe box should be rounded and manufactured with elastic materials to accommodate wide or deformed feet. Schaper et al. [5] and Van Netten et al. [12] concur that such spacing is essential to prevent friction and deformities.

Regarding materials, 25% of the studies emphasise the need for footwear to be flexible, breathable, and free of internal seams. Mohamed et al. [18] and Chicharro-Luna et al. [7] recommend natural leather. Bus et al. [29] underline the importance of flexible tongues and durable materials, while Zhang et al. [23] propose elastic materials to accommodate deformities.

The heel height is mentioned in 18% of the studies, with recommendations ranging from 1.5 to 3 cm to ensure stability without increasing forefoot pressure [5,7,14,18,29]. Likewise, the fastening system (laces or Velcro) appears in 21% of the studies, and is considered by Barwick et al. [19], Bus et al. [29], Ahmed et al. [14], and Schaper et al. [5] as fundamental to securing the foot and ensuring stability within the shoe.

The Scale for the Assessment of Narrative Review Articles (SANRA) was completed to rate the quality of this review. See Appendix A.

## 4. Discussion

Microtrauma, which may lead to ulceration in subjects with DM, represents a significant clinical concern. According to Armstrong et al. [3], up to 34% of individuals with DM develop foot ulcers, with repetitive microtrauma being a notable contributing factor. Abu-Qamar et al. [31] identified footwear as the second most frequent external cause of foot trauma in those patients, accounting for 16.2% of cases.

Footwear type and its features involve multifaceted parameters that are difficult to analyse. Moreover, the correct adjustment of several specific components, such as length, width, materials, and cushioning, presents a challenge to researchers.

In their 2023 publication of the IWGDF Practical Guidelines, Schaper et al. [5] identified ill-fitting footwear as one of the primary causes of foot trauma leading to ulceration. This conclusion is reinforced by Reddie et al. [32], who noted that, in low- and middle-income countries, contrary to the general recommendations for individuals with DM, sandals and flip-flops are the most commonly worn types of footwear.

Populations with lower socio-economic status not only face financial constraints but also tend to have limited health literacy. This is particularly evident among individuals with DM, who generally exhibit lower socio-economic levels compared to non-diabetic populations. Consequently, many are unaware of ulcer prevention strategies, and even when informed, may be unable to afford properly fitted footwear, resorting instead to cheaper alternatives such as sandals and flip-flops.

It should be borne in mind that this phenomenon also occurs in high-income countries with warm climates, such as Spain, where sandals are commonly worn from spring through to autumn due to their breathability, despite being suboptimal for foot protection.

This issue is further supported by Chicharro-Luna et al. [7], who found that most patients with PN did not wear appropriate footwear. Their study revealed that out of 17 subjects with PN, 11 (67.4%) were not using optimal footwear. Although the sample size of PN patients was limited (—only 17 out of 108 participants), the data aligns closely with the findings of Buldt and Menz [6], and offers a valuable insight into the broader reality.

These economic and demographic determinants suggest that international guidelines (largely developed in high-income settings) may not always be feasible or practical in other contexts. Therefore, diabetic foot experts and local healthcare stakeholders should collaborate in order to adapt or redesign preventive recommendations and footwear-related strategies that are both realistic and culturally acceptable.

A positive aspect was highlighted by Malki et al. [25], who found that comfort and fit were the most valued factors among PN patients when selecting footwear. Although their study is limited by its small sample size (24 participants), it is noteworthy that patients prioritise fit and comfort despite their lack of foot sensitivity. This paradox underscores the main challenge in footwear fitting for individuals with DM and PN: their inability to perceive whether the shoe is properly fitted. As described by Malki et al. [25], although patients pay close attention to fit, the reality (as demonstrated by multiple studies) is that the majority wear inadequately fitted footwear.

Buldt and Menz [6] reported that between 10% and 43% of individuals with DM wore shoes that were too short, 23% to 81% wore shoes that were excessively long, and 46% wore shoes that were too narrow. Furthermore, they emphasised that individuals wearing overly tight footwear were five times more likely to develop foot ulcers. These findings are particularly concerning, since they suggest that nearly half of the diabetic population with PN wears shoes that are too narrow, thereby significantly increasing their risk of ulceration.

Abu-Qamar et al. [31] further classified poor fit, incorrect sizing, narrow footwear, and the use of new shoes as sources of microtrauma, and underscored the importance of evaluating all structural elements of footwear.

Regarding shoe length fit, Buldt and Menz [6] recommended a clearance of 10–15 mm between the foot length and the internal length of the shoe. Jones et al. [15] suggested that fit is inadequate if the clearance is less than 2 mm or exceeds 10 mm, although in a subsequent study, they proposed an optimal range of 10–20 mm. The prevailing consensus indicates that deviations outside the 10–15 mm interval may result in improper fit and potential microtrauma.

In terms of width fit, Jones et al. [28] stated that the shoe should match the width of the foot at the metatarsophalangeal joints. Nevertheless, a margin of 2–3 mm on each side is advisable to accommodate dynamic changes in foot volume during gait, as highlighted by Zhang et al. [23], particularly in the presence of deformities such as hallux abductus valgus.

The shoe sole is another critical component. Premkumar et al. [17] noted that cushioning midsoles offer limited stability. Collings et al. [10] emphasised that sole materials should be selected based on offloading requirements. López-Moral et al. [16] and Ahmed et al. [14] concluded that semi-rigid and cushioned soles impair propulsion and increase forefoot plantar pressure, thereby rendering them less suitable for patients with DM and motor neuropathy.

The shoe upper must be flexible, breathable, and free of internal seams. Van Netten et al. [12], Igiri et al. [27], and Chicharro-Luna et al. [7] agreed that it should be made of natural leather or high-quality synthetic textiles. While leather is preferred for its durability and adaptability, synthetic materials may offer similar properties at a lower cost, albeit with reduced longevity. Internal seams should be avoided, as they may cause friction and skin erosion, potentially leading to ulceration.

Collectively, the reviewed studies underscore that improper footwear, whether regarding length, width, sole, or upper, can induce microtrauma and elevate the risk of ulceration in individuals with DM. It is therefore imperative that healthcare professionals, particularly podiatrists, thoroughly assess footwear design and fit in this vulnerable population.

In this context, only a few studies explore footwear selection habits in this population. The factors influencing footwear acquisition and the motivation to change footwear habits when individuals receive specific information regarding its impact on the foot remain unknown. Qualitative research could help towards understanding this issue. Neither do preventive measures appear to be effective, nor is there any common type of footwear that can be considered “protective” for the feet of this population. This term is used by several authors, like Barwick et al. [19], in contrast to so-called “therapeutic footwear,” which tends to be specifically prescribed for particular foot conditions.

We plan to conduct a qualitative study that employs semi-structured interviews, through which people with diabetes can explain the criteria they follow when selecting footwear and the motivations underlying their choices. Moreover, the interview guide will incorporate questions to assess participants’ knowledge of self-care foot practices and their understanding of the types of footwear most appropriate for their condition.

This study has certain limitations, partly due to the variability in the types of designs of the articles included, and also owing to the different levels of evidence. Furthermore, the methodological quality of these articles remains unknown and should be assessed, either through verification guides or by evaluating the risk of bias.

Future studies are needed to establish the ideal type of footwear and the potentially beneficial features for this population in order to guide the prevention strategies that ensure the full collaboration of patients for the preservation of lower-limb health.

## 5. Conclusions

The use of inappropriate footwear remains a widespread issue among individuals with Diabetes Mellitus, constituting a major contributing factor to the development of repetitive microtrauma and subsequent foot ulceration. The limited number of available studies, together with their methodological heterogeneity, underscores the absence of standardized protocols to assess footwear adequacy in this population.

Recommendations concerning proper shoe length, adequate width, rigid soles, and seamless upper materials have demonstrated efficacy in reducing plantar pressure and lowering the risk of ulceration. Nevertheless, adherence to these recommendations remains insufficient.

From a public health perspective, effective prevention strategies must integrate patient education on foot self-care, routine footwear assessment in primary care settings, and programs that facilitate access to appropriate footwear. Additionally, the lack of studies exploring the personal criteria used by individuals with diabetes when selecting their shoes supports the need for research aimed at designing interventions better aligned with patients’ social and economic realities.

## Figures and Tables

**Table 1:** Schematic summary of the databases, search strategies, filters, and results obtained from the search.

Search Strategy	Databases	Search Filters	Results
(“footwear”) AND “Diabetes Mellitus”	PubMed	2016–Present	126
	Web of Science	2016–Present	151
	Dialnet	2016–Present	2
(“Diabetes Mellitus”) AND “Shoes”	PubMed	2016–Present	132
	Web of Science	2016–Present	168
	Dialnet	2016–Present	10
(“diabetic foot”) AND “prevention”	PubMed	2016–Present, Systematic Review	57
	Web of Science	2016–Present	138
	Dialnet	2016–Present	103
(“footwear”) AND “ulcers”	PubMed	2016–Present	145
	Web of Science	2016–Present	212
	Dialnet	2016–Present	10
(prevention) AND (“diabetes”) AND “IWGDF”	PubMed	None	63
	Web of Science	None	97
	Dialnet	None	0

**Table 2:** Description and main outcomes of the articles studied.

Authors, Year, Country	Context	Study Design	Level of Evidence *	Sample Size	Main Outcomes
Bus et al. [13], 2016, Netherlands	Footwear and offloading interventions to prevent and heal foot ulcers and reduce plantar pressure in patients with diabetes	Systematic Review	Ia	80 publications	Rocker-bottom sole designs reduce peak plantar pressure.
Premkumar et al. [17], 2017, India	Footwear in the causation and prevention of foot ulcers in Diabetes Mellitus	Case–control study	III	4800 patients treated over the course of one year	13.6% of ulcers are due to the hardness of the insole. Cushioning midsoles provide limited stability. Rigid and rocker-bottom soles offload the forefoot and help prevent ulcers in this area
Mohamed et al. [18], 2017, United Emirates	To assess behaviours, prevalence of diabetic foot risk factors, and safety of footwear among diabetic patients.	Cross-sectional study	III	74 patients	Although most participants had received education and clinical assessments related to foot care, numerous risk behaviours were observed, such as walking barefoot, wearing shoes without socks, performing self-treatment of calluses, and using inappropriate footwear. Overall, podiatric self-care practices were insufficient, and most patients exhibited one or more predisposing factors for the development of diabetic foot ulcers.
Van Netter et al. [26], 2018, Australia	Develop an updated Australian guideline on footwear for individuals with diabetes.	Narrative Review	IV	None	Length: Should exceed foot length by 1–2 cm. Depth: Sufficient to allow free movement of the toes. Width: Should match the foot’s width across all regions. Height: The higher, the better, as it provides increased stability and reduces forefoot pressure. Sole: Preferably made of rubber. Rocker sole: A rocker sole is preferable; the apex should be positioned proximal to the metatarsophalangeal joints. Heel: Ideally 1.5–2 cm; should not exceed 3 cm in height. Fastening: Lace-up closures are preferable. Upper: Should be made of leather or a similar breathable material that can accommodate foot deformities. Toe box: Must be flexible.
Buldt and Menz [6], 2018, Australia	Determine the prevalence of ill-fitting footwear and examine the association between improperly fitted shoes, foot pain, and foot disorders.	Narrative Review	IV	18 publications	10–43% wore shoes that were too short. 23–81% wore shoes that were too long (difference of 10–15 mm). 46% wore shoes that were too narrow. Foot ulcer patients were 5× more likely to wear overly tight shoes.
Igiri et al. [27], 2019, India	Assess whether footwear contributes to the improvement of plantar pressure distribution in the neuropathic foot.	Narrative Review	IV	11 publications	Toe box: Deep, wide, and square-shaped. Heel: Low, not exceeding one inch in height. Width: Sufficiently wide to prevent friction and the development of blisters. Weight: Lightweight. Fastening: Lace-up or Velcro, with a broad sole to enhance stability. Upper: Made of high-quality leather or fabric. Sole: Rocker sole design.
Barwick et al. [19], 2019, Australia	Identified factors independently associated with inadequate footwear in all inpatient participants, and diabetes and neuropathy subgroups.	Cross-sectional study	III	726 inpatients	Nearly half of patients at risk of foot ulceration were found to wear inadequate footwear, with women being particularly affected. Greater efforts are required to promote adherence to recommended footwear guidelines to prevent ulcer development.
López-Moral et al. [16], 2019, Spain	Analyse the effectiveness of a rigid rocker sole in reducing the recurrence rate of plantar ulcers in patients with diabetic foot.	Randomised Clinical Trial	Ib	51 patients with PN who had recently healed from plantar ulcers.	Rigid rocker soles reduce ulcer recurrence more than semi-rigid soles. 64% lower risk of ulcers with rigid soles vs. semi-rigid soles.
Jones et al. [28], 2019, UK	Review current footwear fitting guidelines and discuss how technology can aid in standardising footwear assessment methods.	Narrative Review	IV	None	Length fit incorrect: <2 mm or >10 mm difference. Width fit correct: Shoe width at metatarsophalangeal joints matches foot width. Width fit incorrect: Shoe is wider or narrower than the foot in this region.
Pérez-Panero et al. [4], 2019, Spain	Review the tiers of evaluation and treatment strategies outlined in clinical practice guidelines focused on diabetic foot or diabetes management.	Systematic Review	Ia	12 publications	The literature recommends the use of therapeutic footwear to reduce shear and friction, supported by level B evidence.
López-Moral et al. [20], 2020, Spain	Identify differences in gait parameters across varying densities of rocker soles, as well as differences in footwear comfort.	Cross-sectional observational study	III	24 patients with diabetes and a prior history of neuropathic foot ulcers.	Rigid soles: Reduce certain gait phases Increase stride velocity and length (vs. semi-rigid soles) Rigid rocker soles: Decrease metatarsophalangeal dorsiflexion Accelerate transition from full stance to toe-off
Bus et al. [29], 2020, Netherlands	Develop and present a bespoke footwear design protocol for patients with moderate- to high-risk diabetes and PN.	Narrative Review	IV	None	A minimum of 1 cm between the internal length of the shoe and the length of the foot. Toe box sufficiently high. Absence of internal seams. Fastening with laces or Velcro. Adequate shock absorption. Durable and lightweight materials. Should provide stability and feature a distal rocker sole. In cases of edema, a low-cut design is recommended; otherwise, a high-cut design with padding in this area is preferable. Flexible tongue. Heel height of 1.5–2 cm for men and 2.5–3 cm for women.
Collings et al. [10], 2020, UK	Identify the optimal design features of footwear and insoles that effectively offload the plantar surface of the foot to prevent ulceration in individuals with diabetic peripheral neuropathy.	Systematic Review and Meta-analyses	Ia	54 publications	Rigid soles: Reduce metatarsal head pressure by 30–50% (limit joint movement). Rocker sole: Optimal for reducing plantar pressure; design (height, position, angle) must be individualised. Sole material: Should be selected and combined based on specific offloading needs
Zwaferink et al. [21], 2020, Netherlands	Evaluate the effect of data-driven personalised footwear concepts on plantar pressure relief for the prevention of diabetic foot ulceration.	Cross-sectional observational study	III	24 diabetic neuropathic patients at high risk of ulceration.	The importance of footwear design for patients with Diabetes Mellitus lies in its foundation on biomechanical studies, plantar pressure measurements, and systematically reviewed, evidence-based scientific research.
Ahmed et al. [14], 2020, Australia	Summarise and evaluate the evidence on footwear and insole features that reduce pathological plantar pressures and the occurrence of neuropathic diabetic foot ulcers in the plantar forefoot of individuals with diabetic neuropathy.	Systematic Review	Ia	25 publications	Rigid rocker soles (Apex at 52% of sole length, 20° rocker angle, 95° apex angle): Achieve plantar pressures <200 kPa in 71–81% of cases. Pivot under metatarsal heads + rigid materials: Superior forefoot offloading vs. semi-rigid rocker soles. Recommendations for diabetic patients with neuropathic ulcers: High-cut footwear (above ankle). Stiff tongue and upper shaft. Rigid rocker soles with proximal pivot point.
Chatzistergos et al. [22], 2020, Malta	Investigate the potential benefits of using footwear with optimised cushioning on individuals with DM and PN.	Observational study	III	15 individuals (6 men, 9 women) who presented diabetic foot at the general hospital of Malta.	The use of cushioning with stiffness specifically optimised for an individual patient significantly reduces plantar pressure compared to using the same material uniformly across all patients.
Jones et al. [30], 2020, UK	Evaluate the measurement gap in footwear used by individuals with DM.	Narrative Review	IV	8 publications	There is no exact consensus regarding the ideal gap between foot length and the internal length of the footwear. However, most authors suggest an optimal range between 1 cm and 1.5–2 cm.
Abu-Qamar et al. [31], 2020, Australia	Examine the sources of external trauma that contribute to the development of foot ulcers in individuals with diabetes and the outcomes of such ulcers.	Narrative Review	IV	45 publications	Shoes are the second most frequent cause of foot trauma, just behind puncture or penetrating injuries, and account for 16.2% of all trauma types. Contributing factors include poor fit, incorrect size, tight or constrictive footwear, and new shoes.
Chicharro-Luna et al. [7], 2021, Spain	Determine whether patients with and without PN use appropriate footwear.	Cross-sectional observational study	III	108 patients with DM.	Most patients with peripheral neuropathy (*n* = 17) do not wear appropriate footwear (11 deemed inadequate). Footwear is considered optimal if it meets the following criteria: Upper made of natural leather. Absence of internal seams. Dorsal fastening with Velcro or laces. Stable heel no higher than 3 cm. Rounded or circular toe box with sufficient width to allow toe movement. Flexion of the shoe at the metatarsophalangeal joints.
Jones et al. [15], 2021, UK	Evaluate the efficacy, methodological coherence, and potential for refinement of pressure thresholds in footwear.	Systematic Review	Ia	21 publications	Five pressure thresholds in footwear that reduce the risk of ulcers were identified: Pressures below 200 kPa. Sustained pressure below 35 kPa. Risk of ulceration. Shoe size. Foot region.
Zhang et al. [23], 2021, China	Analyse the deformities of diabetic foot under three different weight-bearing conditions using foot scanning technology that enables efficient simultaneous examination of the dorsal and plantar surfaces of the foot.	Observational Study	III	The feet of 48 patients with Diabetes Mellitus were scanned.	Foot structure changes in static and dynamic conditions; footwear/materials should accommodate this for better fit. Wide feet: Recommend wider toe box and stretchable upper materials. Gender influences footwear design for diabetic patients.
Luo et al. [11], 2022, China	Examine whether specialised therapeutic footwear could reduce the incidence of foot ulcers.	Systematic Review and meta-analyses	Ia	8 publications	Specialised therapeutic footwear with offloading features significantly reduces the incidence of foot ulcers.
Malki et al. [25], 2023, Netherlands	Determine the factors considered important for the use of therapeutic footwear among different groups of individuals with DM and PN.	Qualitative Study	IV	24 patients were divided into three groups based on disease severity.	Comfort and fit were the highest-rated factors by participants with PN.
Sousa et al. [24], 2023, Portugal	Develop innovative footwear to prevent ulceration, specifically a shoe and insole equipped with sensors that will enable monitoring of pressure, temperature, and humidity parameters.	Observational Study	III	919 diabetic patients from a selected primary care organisation in Portugal.	Fit of the footwear (length, width, and depth) General characteristics (material, design, weight, etc.) Movement properties (density, stability, etc.) Cushioning
Reddie et al. [32], 2023, USA	Summarise the current knowledge regarding footwear used in low- and middle-income countries.	Narrative Review	IV	25 publications	In low- and middle-income countries, the most used types of footwear are sandals and flip-flops, which contradict the general footwear guidelines for patients with Diabetes Mellitus.
Schaper et al. [5], 2023, Netherlands	Describe the basic principles of prevention, classification, and management of diabetes-related foot disease based on the seven IWGDF guidelines.	Narrative Review	IV	None	Inappropriate footwear is one of the main causes of foot trauma that can lead to ulceration. There should be 1–2 cm of space between the length of the foot and the internal length of the shoe. The width of the shoe at the level of the metatarsophalangeal joints should match the width of the foot. The height of the toe box should be sufficient to allow toe movement.
Van Netten et al. [12], 2024, Netherlands	Evaluate the efficacy of interventions aimed at preventing foot ulcers in individuals with diabetes who are at risk of developing such ulcers.	Systematic Review and meta-analyses	Ia	51 publications	Both the use of therapeutic footwear and insoles reduce plantar pressure and decrease the incidence of initial and subsequent ulcerations.

* Levels of evidence proposed by the US Agency for Healthcare Research and Quality [33].

**Figure A1:**
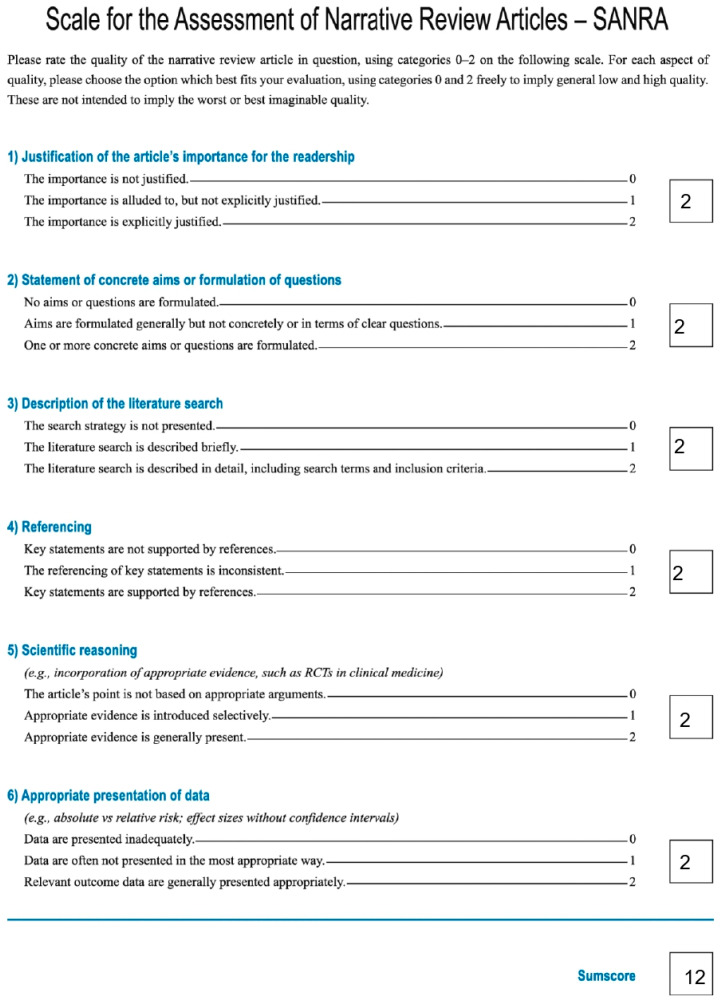
Scale for the Assessment of Narrative Review Articles—SANRA.

## Abbreviations

The following abbreviations are used in this manuscript:

DM	Diabetes Mellitus
PN	Peripheral Neuropathy
PAD	Peripheral Arterial Disease
IWGDF	International Working Group on the Diabetic Foot
